# From *Who* experiences stress to *When* It Occurs: Revisiting Lazarus’ transactional model of stress using an ecological momentary assessment framework

**DOI:** 10.1371/journal.pdig.0001716

**Published:** 2026-09-15

**Authors:** Dennis John, Hannah van Alebeek, Rüdiger Pryss, Carsten Vogel, Johannes Schobel, Teresa O’Rourke, Anne-Kathrin Helten, Jennifer Apolinário-Hagen, Thomas Probst

**Affiliations:** 1 Institute for Applied Research and Evaluation, Lutheran University of Applied Sciences, Nuremberg, Germany; 2 Division of Psychotherapy, Department of Psychology, University of Salzburg, Salzburg, Austria; 3 Institute of Clinical Epidemiology and Biometry, University of Würzburg, Würzburg, Germany; 4 Institute of Medical Data Science, University Hospital Würzburg, Würzburg, Germany; 5 DigiHealth Institute, Neu-Ulm University of Applied Sciences, Neu-Ulm, Germany; 6 Faculty of Psychology, University of Vienna, Vienna, Austria; 7 Social and Environmental Medicine, Faculty of Medicine, Institute for Occupational, Heinrich Heine University Düsseldorf and University Hospital Düsseldorf, Düsseldorf, Germany; 8 Center for Digital Medicine, Heinrich Heine University Düsseldorf, Düsseldorf, Germany; University of Salford, UNITED KINGDOM OF GREAT BRITAIN AND NORTHERN IRELAND

## Abstract

According to the Transactional Model of Stress, stress responses arise from one’s perceptions of situational relevance (primary appraisal) and coping resources (secondary appraisal) as being insufficient. Although the model assumes moment-to-moment within-person variability, most research evidence stems from cross-sectional study designs. Therefore, this study applies Ecological Momentary Assessment (EMA) to test the dynamic interplay between primary and secondary appraisals and fluctuations in perceived stress. We used EMA data from the TrackYourStress platform to examine both interindividual (“who”) and intraindividual (“when”) associations between primary and secondary appraisals and perceived stress. Bayesian multilevel models were applied to 2,908 observations from 164 participants. These models showed that higher appraisal of situational relevance was associated with increased stress, whereas higher appraisal of coping resources was associated with lower stress levels. Importantly, the association between relevance appraisals and stress was stronger under low coping resource appraisals. Findings were present at the inter- and intraindividual level. These findings support the model’s dynamic assumptions and highlight that appraisals not only account for interindividual differences in stress levels but also for moment-to-moment variation in stress responses in daily life.

## Author summary

Stress is not simply the result of what happens to us. The same situation can feel overwhelming to one person and manageable to another, and it may distress us on one day but not the next. Decades ago, Richard Lazarus proposed that stress depends on two personal judgements: how relevant or demanding a situation seems, and whether we feel we have the resources to cope with it. Although this influential model assumes that stress shifts from moment to moment, most studies have tested it only once per person, making these dynamics hard to capture. We used the smartphone app TrackYourStress to ask 164 people about their stress and their appraisals repeatedly during everyday life, collecting 2,908 reports. This let us separate stable differences between people (“who feels more stressed”) from momentary changes within the same person (“when someone feels stressed”). Situations judged as more relevant were linked to more stress, while feeling well-resourced was linked to less. Relevance stressed most when coping resources felt low — both between and within people. These findings support Lazarus's model in daily life and point to new opportunities for smartphone-based tools that monitor and support people's stress in real time.

## Introduction

### Stress and the transactional model of stress

**Fig 1:**
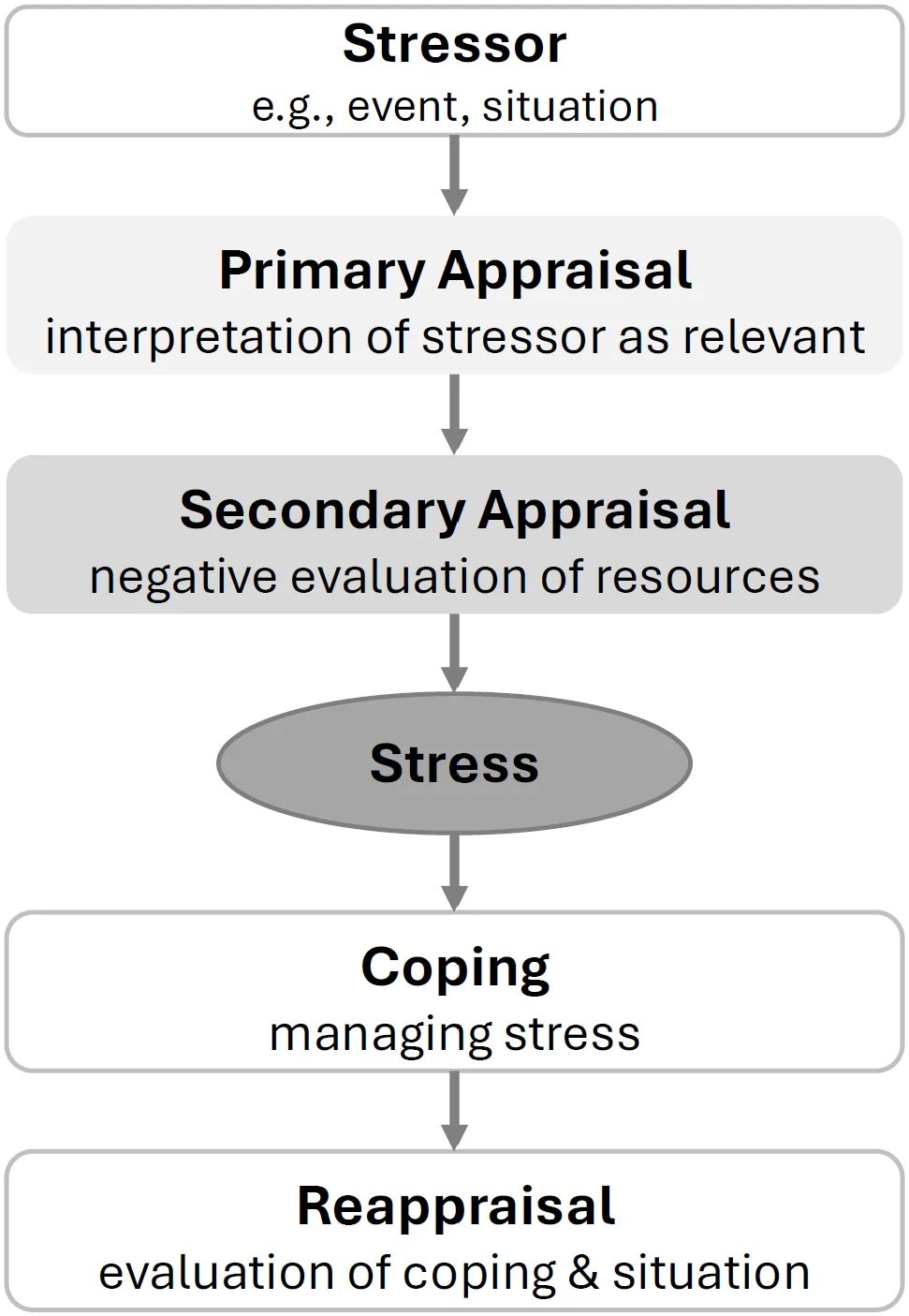
The Transactional Model of Stress.

Why do two people facing the same situation (e.g., medical consultation or therapeutic intervention) sometimes respond differently? And why does a similar situation elicit stress at one time but not at another, even within the same individual? According to the Transactional Model of Stress proposed by Lazarus [1] and Lazarus and Folkman [2], such differences in stress responses arise not primarily from the objective characteristics of situations, but from individuals’ cognitive appraisals of the situations. Situations in this context can also be internal stimuli (e. g., elevated heart rate leading to stress in some people, but not in others). The model proposes two key stages of appraisal: primary appraisal, referring to the evaluation of a situation’s relevance and potential implications, and secondary appraisal, which concerns the individual’s perceived ability to manage or cope with the situation. Stress arises when a situation is perceived as relevant to personal well-being and the perceived demands of the situation are judged to exceed one's own resources to cope with or overcome the situation (see Fig 1).

In primary appraisal, individuals evaluate whether a situation is relevant to their well-being and determine its potential implications. This process includes judging whether the situation is relevant for oneself or not. According to the Transactional Model of Stress, situations appraised as irrelevant typically lead to minimal stress responses, underscoring that subjective interpretation is the first critical step in determining whether a stress response is triggered at all [2]. Importantly, primary appraisal is not a simple dichotomous decision; rather, it involves a nuanced evaluation shaped by personal goals, values, prior experiences, and the situational context as well as stress-related mindsets [3]. Two individuals encountering the same situation may therefore attribute different meanings to it, leading to divergent stress reactions [4]. For example, in medical consultations one patient may interpret a discussion about a possible diagnosis as a serious threat, while another appraises the same conversation as informative and controllable [5,6]. Even within the same person, the meaning attributed to a similar situation can fluctuate depending on current emotional and bodily states, competing demands, or shifting priorities. Moreover, appraisals may also be shaped by contextual factors outside the person, such as concurrent or cumulative stressors, which can alter how a situation is evaluated at a given moment.

In secondary appraisals, individuals evaluate their coping resources available to manage the situation that has already been judged as relevant during the primary appraisal [2]. Coping is defined as the ability to manage a situation that an individual perceives as relevant [7,8] and can vary between individuals as well as within individuals over time [9]. The original model distinguishes problem-focused coping (actively changing a stressful situation) from emotion-focused coping (managing its emotional consequences). More recent perspectives, such as dual-processing theory [10,11], additionally emphasize coping flexibility, that is, adjusting coping strategies based on their prior effectiveness rather than relying on fixed ‘adaptive’ versus ‘maladaptive’ categories of coping.

Interindividual differences in coping have been documented for demographic factors such as gender [12,13]. At the same time, intraindividual differences must also be considered, as coping responses may fluctuate depending on the frequency, intensity, or accumulation of potential stressors across time [9,14].

Ultimately, the interplay between these primary and secondary appraisals shape both the coping response and the resulting intensity of stress level [2].

The Transactional Model of Stress [1,2] furthermore describes how individuals reappraise situations and available coping resources based on the perceived effectiveness of utilized coping strategies. Depending on these re-evaluations, the originally appraised situation may shift, for example, from a threat appraisal to a challenge appraisal or vice-versa.

Consequently, both perceived stress and coping responses should be conceptualized not as static reactions, but as dynamic processes that evolve through continuous reappraisal of situations, coping resources, and coping efficacy [15]. Although such within-person, iterative appraisal processes are central to the model, they cannot be sufficiently investigated in cross-sectional approaches [16].

### Between- and within-person variability in stress

Stress levels exhibit marked variability both between and within individuals [17]. Interindividual differences arise from factors such as genetic predispositions, developmental experiences, and personality traits [9,18,19]. Intraindividual fluctuations, by contrast, are driven by momentary conditions, including the severity, intensity, and frequency of daily stressors [9]. Given this dynamic nature of stress, the assessment of both inter- and intraindividual aspects is of paramount importance for understanding stress-related risk pathways that contribute to the development of mental and physical health problems. Since its introduction, the Lazarus Transactional Model of Stress has provided a foundational framework for identifying the cognitive mechanisms, particularly appraisal processes that moderate stress responses. Recognizing these mechanisms has informed the development of interventions aimed at reducing stress-related burden. In fact, stress management programs play a critical role in helping individuals modify maladaptive appraisal patterns and strengthen their coping resources [20,21]. Interventions such as cognitive-behavioral techniques, mindfulness-based strategies, and resilience training have been shown to reduce stress by promoting more adaptive interpretations of stressful situations [22,23].

### Ecological momentary assessment

Collecting data in real-world settings is advantageous because behaviors and experiences can be assessed in their typical context and individual contextual variables such as time of day or time budget can be taken into account [24]. Contrary to stress protocols in laboratory settings or under controlled conditions, a method of data acquisition that enables the generalization of results to real-world contexts is Ecological Momentary Assessment (EMA), which is defined by the assessment of data in real-world environments, in real time, and over multiple measurement time-points [25,26]. An advantage of EMA studies is that they enable the measurement of coping in real time, so that any distortions caused by retrospective assessments can be avoided. For example, people tend to be more likely to remember situations that are more relevant to them personally, happened more recently, or are unusual, which can bias retrospective reports [27]. By including repeated measures, within-subject changes in behavior and experience over time can be investigated [25]. In the 1980s, Stone and Neale conducted one of the earliest EMA-related studies regarding coping to create an assessment tool for daily coping [28]: Within a bigger longitudinal study, they sent out questionnaires to 120 married couples, which filled these out over a period of 21 days. More recently, with the increasing use of smartphones and other electronic devices in the general population, EMA designs have become more widely applied in various areas of stress research [4,29]. With the advance of digitalization, smartphone-based mobile health (mHealth) apps have become popular, particularly during COVID-19 [30,31]. They are a low-threshold option of stress and mental health assessments, as they require little effort to engage with, can be used anywhere and have the potential to reach large numbers of people [32]. Such mHealth apps facilitate the integration of instruments (i.e., questionnaires) into daily life, which enables the assessment of dynamic stress and coping processes over time with EMA designs in contrast to retrospective assessments, which cannot take such dynamic changes into account.

### The current research

Research testing the Transactional Model of Stress often shows a discrepancy between its theoretical assumptions and the manner in which the model is operationalized in empirical studies. Although the model conceptualizes appraisals as dynamic processes, most empirical studies rely on cross-sectional designs (see below). Such approaches can only assess whether individuals who generally appraise situations as more relevant and their coping resources as lower than others report stronger stress responses. However, they cannot show whether certain situations that are considered more relevant than the typical starting point for a particular person, or moments in which a person's coping resources are considered to be lower than usual, are associated with increased stress. Capturing these within-person fluctuations requires repeated-measures approaches such as EMA. While EMA designs generally have the advantage of assessing experiences in real time, thereby reducing recall bias, their primary value lies in the fact that they enable the dissociation of within- and between-person effects through appropriate centering techniques. This approach allows to test both, whether the proposed appraisal mechanism account for (1) interindividual differences in stress responses (i.e., who is prone to stress) and (2) intraindividual variations, specifically whether fluctuations in primary and secondary appraisals within an individual determine when this individual experiences stress and when not.

So far, empirical studies on the transactional model’s core assumptions have mostly relied on cross-sectional designs and on specific populations such as patients [33,34] and caregivers [35]. In addition, research examining the predictions of the Transactional Model of Stress frequently reveals a discrepancy between its theoretical assumptions and their methodological operationalization [34]. One of few repeated-measures studies in cancer patients provided only limited support for longitudinal links between changes in appraisal and emotional outcomes from baseline to 3- and 6-month follow-up [36]. On the other hand, stress level is among the most extensively assessed constructs in EMA research. A large body of work has used EMA to capture momentary stress and stress reactivity in daily life, documenting substantial within-person fluctuations in perceived stress and their associations with affect, behavior, and health [29,37–39]. This literature has also produced validated momentary stress measures tailored to intensive longitudinal designs [40]. More recently, EMA studies have been implemented to evaluate the Transactional Model’s dynamic, within-person assumptions in daily life contexts. For example, increased stress-intensity appraisals have been shown to predict subsequent negative affect and binge eating [41]. Challenge appraisals were found to be associated with greater use of subsequent problem-focused coping, whereas threat appraisals predicted emotion-focused strategies [42]. In a daily-diary study, higher primary and lower secondary appraisal of the day's main stressor were each associated with greater negative affect [43]. Within this maturing field, however, few studies have simultaneously operationalized both primary (relevance) and secondary (coping resource) appraisals as predictors of momentary stress, and, to our knowledge, none has tested their transactional interaction while dissociating within- from between-person effects.

### Research questions (RQ)

RQ1 - The “Who”: Do individuals with generally higher relevance appraisals and lower coping resource appraisals experience more stress?
RQ2 - The “When”: Are moments with higher relevance appraisals and lower coping resource appraisals associated with higher momentary stress?

## Methods

### Ethics statement

Approval for this study was granted by the Ethics Committee of the University of Würzburg, Germany (approval no. 103/22-sc) and the study was conducted in accordance with the Declaration of Helsinki. All participants provided electronic informed consent prior to study participation.

### Measures and EMA procedures

**Fig 2:**
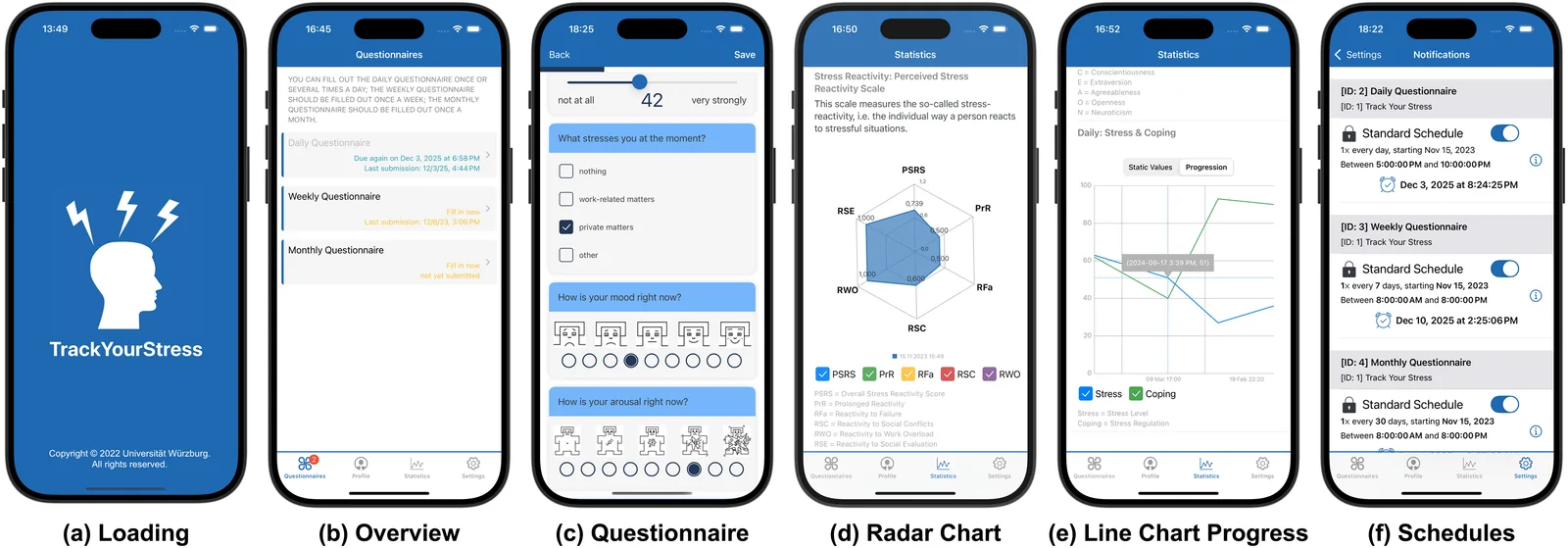
The TrackYourStress mHealth EMA platform. *Note.* This figure displays TrackYourStress screenshots: (a) the loading screen, (b) the landing page with a questionnaire overview, (c) an open questionnaire, (d) the data visualization page with a radar chart, (e) the data visualization page displaying progress with a line chart and (f) the schedule overview page for each repeating questionnaire. Interface icons of the mobile app are derived from native iOS system elements, Apple SF Symbols, and selected Icons8 assets (https://icons8.com). *Abbreviations:* EMA = Ecological Momentary Assessment; mHealth = mobile health.

TrackYourStress is an mHealth EMA platform, which offers a website (registration and account management), an Android and iOS mobile app, a MariaDB relational database for the central repository to store the collected data, and a backend component. The latter offers a sophisticated Representational State Transfer (“RESTful”) application program interface (API) that is used by the mobile apps [17]. The mobile application includes, among other features, a loading screen, a landing page with an overview of available questionnaires, interfaces for completing open questionnaires, data visualization pages (e.g., radar charts), and schedule overview pages for each repeating questionnaire (see Fig 2). In the Supporting Information, S1 Text, S1 Fig, S2 Text, S2 Fig, S3 Text, S3 Fig, S4 Text, S4 Fig, S5 Text, S5 Fig illustrate how the different procedures are implemented in the TrackYourStress app (e.g., registration process, receiving and responding to notifications, or setting options for notifications).

The applied data collection procedure for all TrackYourStress users is as follows: First, they register through the website or the mobile apps. Second, users have to fill in the registration questionnaire once. This includes demographic data (i.e., gender and date of birth). Third, the continuous mobile EMA procedure starts. In this phase, users have the option to select a predefined notification schema or fill in the questionnaires as they like. If they select a predefined notification schema, this schema determines the frequency and in what way (i.e., fixed or random points in time) daily, weekly, and monthly questionnaires are applied. For each questionnaire, users can, however, modify the predefined notification schedule by defining a preferred timeframe and the desired number of reminders within these periods. Each time a notification appears, the user may click on it to start the mobile app (if not already running), and the respective questionnaire is then shown directly to the user. It is also always possible for users to complete the questionnaires without notifications (i.e., if they are particularly stressed and want to report it). After completion, the results are transferred to the server through the RESTful API if the mobile app is online, otherwise the results are locally stored until the device is connected again. For a detailed technical description of these features, we refer to [44,45]. The website (www.trackyourstress.org) and the RESTful API of TrackYourStress are publicly released, and the smart mobile app TrackYourStress is distributed through the official mobile app stores from Apple and Google. For the present study, the daily questionnaire was the relevant instrument. During registration users decided whether to allow notifications. If notifications were enabled, participants received one reminder per day at a self-selected time; if they were disabled, participants could still complete the daily questionnaire on their own initiative at any time. Although users were thus able to provide more than one entry per day, only the first daily assessment was included in the present analyses.

For the current study, Items 1, 8, and 9 of the daily questionnaire were examined to test the Transactional Model of Stress. The items of the daily questionnaire can be obtained from Table 1. Three basic user interface elements were implemented to answer a question by a TrackYourStress user. First, we implemented sliders, which represent the Visual Analogue Scales (VAS). Second, only for Question 5 (“What stresses you at the moment?”), we implemented a user interface element called Category, which, in turn, shows the following four categories to a TrackYourStress user: nothing, work-related matters, private matters, and other. The user can then select all categories, which are currently stressful for him or her. Third, we implemented a user interface element to provide the so-called Self-Assessment Manikins [46] (SAM) on Android and iOS.

**Table 1:** Items of the TrackYourStress Daily Questionnaire.

Item No.	Question	Scale	Range
1	How high is your momentary stress level?	VAS^a^	0-100: Very low - very high
2	How well can you control your momentary stress level?	VAS	0-100: Not at all - very well
3	How strongly are you experiencing your momentary stress level as negative/impairing?	VAS	0-100: Not at all - very strongly
4	How strongly are you experiencing your momentary stress level as positive/beneficial?	VAS	0-100: Not at all - very strongly
5	What stresses you at the moment?	C^b^	4 categories: nothing, work-related, personal, other
6	How is your mood right now?	SAM^c^	0-8: very bad - very good
7	How is your arousal right now?	SAM	0-8: low arousal - high arousal
8	How important is the situation that you are experiencing in this moment for you personally?	VAS	0-100: Not at all - very important
9	How would you assess your resources to manage the situation that you are experiencing in this moment?	VAS	0-100: not sufficient - absolutely sufficient

Note. ^a^VAS = Visual Analogue Scale. ^b^C = categories. ^c^SAM = Self-Assessment Manikins [46].

*Perceived Stress Level* was operationalized using Item 1 as the outcome/dependent variable (M = 36.15, SD = 27.29). As repeated-measures predictors, *Primary Appraisal* was operationalized using Item 8 (M = 59.99, SD = 27.45) and *Secondary Appraisal* was operationalized using Item 9 (M = 70.40, SD = 27.29). Table 2 indicates how much of the variance in these variables can be attributed to interindividual differences (i.e., the “Who”) and how much remains at the intraindividual level (i.e., the “When”) and it indicates whether the proportion of interindividual variance meaningfully deviates from 50%, which corresponds to an equal amount of variance at both levels based on the intraclass correlation coefficient (ICC). Because each momentary construct was assessed with a single item, this intraindividual component comprises genuine within-person (“when”) fluctuation together with occasion-level measurement error; it therefore represents an upper bound on true momentary variability. The ICC decomposition is used primarily to justify the multilevel modelling approach rather than to quantify the amount of genuine “when” variation.

**Table 2:** Variance Decomposition at the Interindividual Between-person and Intraindividual Within-person Level.

	Variance Decomposition		Evidence for deviation from equal variance partition (ICC = 50%)	
**Variable**	**Interindividual**	**Intraindividual**	**95% HDI (ICC)**	**PD (ICC > 50%)**
Stress	35.58%	64.42%	[29.59%, 41.64%]	0%
Primary Appraisals	49.94%	50.06%	[44.02%, 56.05%]	49.12%
Secondary Appraisals	54.95%	45.05%	[49.13%, 60.98%]	94.93%

*Note.* The proportion of interindividual variance was based on the ICC derived from three Bayesian multilevel models predicting stress, primary and secondary appraisals, whereas the proportion of remaining intraindividual variance was calculated as its complementary share (1 − ICC). The 95% HDI reflects the posterior uncertainty of the ICC; if it does not include 50%, this is taken as evidence for an unequal variance partition. The PD represents the proportion of the posterior distribution above 50%, that is, the probability that interindividual variance exceeds intraindividual variance. *Abbreviations:* ICC = intraclass correlation coefficient; HDI = highest-density interval; PD = probability of direction.

### Study design

This was a retrospective cohort study of an adult population, based on the analysis of data from the TrackYourStress mHealth Ecological Momentary Assessment (EMA) platform collected between the 8^th^ January 2018 and the 22^nd^ July 2025. Data collection for the TrackYourStress project occurred in three phases among an adult population.

(a) The first data collection phase was conducted between July 2018 and January 2019 as part of an Ecological Momentary Assessment (EMA) study using the TrackYourStress EMA platform and mobile apps [17,47]. Three research assistants from the FOM University of Applied Sciences in Augsburg and Munich recruited participants during this period. A convenience sample was obtained through the universities as well as the students’ personal and professional networks. Individuals were invited via email and could register for study participation after providing informed consent. Participants were asked to use the TrackYourStress app daily for four weeks and did not receive any compensation. As the TrackYourStress mobile application was not fully released via the official stores at this time, we used a private installation routine to install the app on the participants’ smartphones.
(b) Beginning in 2023 until now, the TrackYourStress app can be downloaded for free in the Apple App Store and Google Play Store. Potential participants could download the app independently, provide informed consent within the app, and register for study participation. Again, no compensation was provided for app use during this phase. Participation in this phase was motivated by users’ own interest in self-monitoring their stress, supported by the app's feedback on their personal stress trajectories over time.
(c) From 2023 to 2025, data collection took place at the Lutheran University of Applied Sciences and was supported by cooperating partners, such as the Institute for Occupational, Social and Environmental Medicine at the Heinrich Heine University Düsseldorf as part of the research project *Relaxed Studying*. Students were invited to participate by downloading the TrackYourStress app from the app stores. Recruitment involved university-wide email announcements sent to all enrolled students and in-class invitations in seminars and lectures, during which a research assistant briefly introduced the study and provided a QR code linking to the Google and Apple App stores. Students could download the App, register, and provide informed consent. Within the study protocol students were asked to use the TrackYourStress app daily for at least two weeks. For students recruited via seminars and lectures, participation was additionally tied to their involvement in the respective courses. In contrast to the earlier phases, students could enter a raffle for one of eight €25 vouchers for an online store (https://www.avocadostore.de) after completing participation.

### Analysis

#### Pre-processing.

We first removed 27 duplicated observations (likely submitted twice due to technical device errors). Finally, three participants reported impossible birthdates (e.g., dates in the future); thus, their age at study start was set to missing. No further observations were excluded.

To disentangle between-person variance (i.e., the “who”; interindividual differences) from within-person variance (i.e., the “when”; intraindividual differences), both repeated-measures predictors, namely the relevance and coping resource appraisals, were person-mean centered (i.e., z-standardized within individuals). Each person-mean was additionally z-standardized on the overall sample mean. This procedure yielded four variables: two between-person variables capturing interindividual differences, namely if an individual generally appraises situations as more or less relevant, or appraises more or fewer coping resources, compared to others; and two within-person variables capturing intraindividual differences, namely fluctuations around each individual’s own typical level (i.e., moments when a given person appraises situations as more or less relevant than usual, or appraises more or fewer coping resources than usual), independent of their overall standing relative to other participants. Because all variables were standardized, a value of 1 for between-person predictors represents a score that is 1 SD higher (or lower, if negative) than the samples mean for that variable, and a value of 1 for within-person predictors represents a score that is 1 SD higher (or lower, if negative) than this person’s own mean across time points.

#### Models.

To examine how stress levels are influenced by both interindividual and intraindividual differences in primary and secondary appraisals, we specified two models. The first predicted stress levels across timepoints using the interaction between between-person primary and between-person secondary appraisals. The second predicted momentary stress levels using the interaction between within-person primary and within-person secondary appraisals. Because observations in the within-person model were nested within individuals, we used a multilevel model with random intercepts, slopes, and interactions per participant. This maximal random-effects structure provided the best model fit, as indicated by leave-one-out cross-validation. In the between-person model, we included age and gender to test whether the findings remain robust when additional between-person covariates are considered. These additional checks were not applied to the within-person model, as within-person effects are unaffected by between-person variables due to centering.

Both models were estimated within a Bayesian framework. This has the advantage that we can quantify *continuous* evidence, and thus can not only quantify the evidence for an effect but also for no effect (i.e., null result). Uninformative default priors from the *brms* package [48] were used to avoid imposing assumptions on effect sizes, ensuring that the likelihood from the data predominantly shaped the posterior distributions. All models converged without divergent transitions, with Rhat values < 1 and effective sample sizes (ESS) > 400. Posterior distributions are summarized in the main manuscript using 95% highest-density intervals (HDIs), which serve as a Bayesian analogue to the conventional 95% interval in frequentist statistics (noting, however, that this threshold is arbitrary). We additionally report the probability of direction (PD), which quantifies the proportion of the posterior distribution above zero and provides continuous evidence regarding effect direction. To assess practical significance, we defined a Region of Practical Equivalence (ROPE) around zero, representing effects considered negligible. Following common recommendations [49,50], the ROPE was set to -0.1 to 0.1 for standardized parameters (i.e., half of a small effect size). Effects were interpreted as follows: if more than 95% of the posterior distribution fell inside the ROPE, the effect was considered practically equivalent to zero (i.e., null result); if between 5% and 95% fell inside the ROPE, the evidence was deemed inconclusive; and if less than 5% fell inside the ROPE, the effect was considered practically significant, indicating more than 95% certainty that the effect was non-negligible.

### Transparency and openness

The sample size was determined by the number of participants that logged into the TrackYourStress App from its development until the start of analyses, with no additional exclusions. As the dataset is continuously updated, previous publications have utilized parts of it [12,17], but not the variables relevant in the context of the current study. The analysis plan was pre-registered before the analysis for the current study were performed and can be accessed via Open Science Framework (link: https://osf.io/4ajrs). However, this study’s design and analysis were not pre-registered before data collection started.

## Results

### Sample characteristics

After removing test users, 211 participants remained. Among these 211, 47 provided only a single observation and were therefore excluded because they exhibited no within-person variability (see decomposition of variance, Table 2). This resulted in a sample of 164 participants for this study. The sample description is provided in Table 3 as is the comparison between included (n = 164) and excluded study participants (n = 47) in baseline variables (gender, age, stress at registration). Included and excluded participants did not significantly differ in age and gender, but those who only provided one entry (excluded participants) had higher stress levels at this first entry (p < .001) than those who provided at least two entries (included participants).

**Table 3:** Sample Description and Statistical Comparisons Between Included and Excluded Participants in Baseline Variables.

	Sample			
Variable	Included (n = 164)	Excluded (n = 47)	t/ χ2 (df)	p value
Female (%)	60.36%	57.45%	0.04 (1)^a^	.719
Age (years), mean (SD)	33.20 (11.92)	31.96 (11.96)	1.04 (42.14)^b^	.304
Stress level at registration, mean (SD)	37.60 (18.16)	54.02 (26.55)	4.20 (47.66)^b^	< .001

*Note***.** ^a^Test of Given Proportions ^b^Welch Two Sample t-test. *Abbreviations:* SD = standard deviation; n = number of participants; df = degrees of freedom; t = t statistic; χ^2^ = chi-square statistic

The final sample remained active in the app for an average of 203.58 days (*SD* = 146.07 days; range = 48 minutes to 515.86 days) and during that time they on average provided 17.73 app entries (*SD* = 13.52; range = 2–126 entries), resulting in 2,908 datapoints. Of these entries, participants reported on 1,091 that nothing stressed them, on 1,058 that they were stressed by work-related matters, on 1,056 by private matters, and on 403 by other factors. Participants could select more than one reason per entry. Because participants varied substantially in the number of entries provided and the duration they stayed in the app, we examined the robustness of our findings across multiple thresholds for both data volume and tracking duration (i.e., successively excluding participants with many prompts or long duration from first to last entry; see Supporting Information S6 Text, S6 Fig, S7 Fig, S7 Text, S8 Fig). Findings can be considered relatively robust, even for the least robust effect, significance was maintained until more than 55% of the sample had been removed. We additionally examined whether results were robust across recruitment phases by including recruitment phase as a moderator; the effects were relatively robust and did not differ by phase (see Supporting Information S8 Text, S1 Table).

### Do stress levels depend on primary and secondary appraisals?

An overview of the results is depicted in Table 4.

**Table 4:** Results of the Between and Within-Person Models.

	Between-Person Model (the „Who“)				Within-Person Model (the „When“)			
*Predictors*	*b*	*95%HDI*	*PD*	*ROPE*	*b*	*95%HDI*	*PD*	ROPE
Primary Appraisals	**.24**	**[.12, .35]**	**99.99%**	**0.90%**	.11	[.06, .17]	99.98%	31.80%
Secondary Appraisals	**-.64**	**[-.75, -.53]**	**100%**	**0%**	**-.39**	**[-.44, -.34]**	**100%**	**0%**
Primary × Secondary	-.15	[-.25, -.06]	99.94%	13.81%	-.07	[-.10, -.03]	99.98%	97.83%

*Note*. Bold values denote effects considered practically meaningful. *Abbreviations:* b = standardized regression coefficient; 95% HDI = 95% highest-density interval; PD = probability of direction; ROPE = Region of Practical Equivalence.

#### RQ1 - The “Who”: Do individuals with generally higher relevance appraisals and lower coping resource appraisals experience more stress?

**Fig 3:**
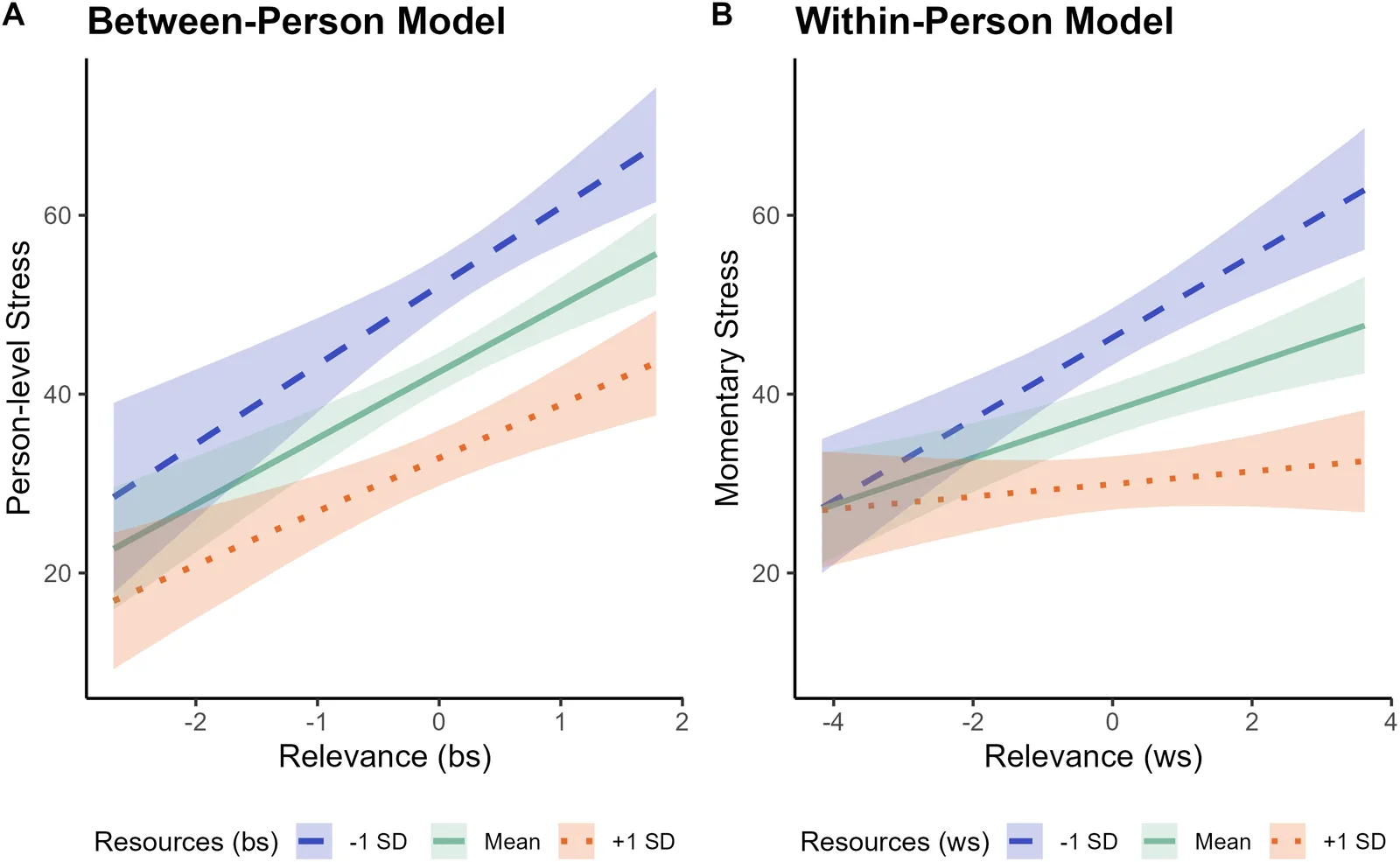
Associations between relevance (primary) appraisals and stress depending on coping resource (secondary) appraisals for between-person (A) and within-person (B) analyses. In each panel, the x-axis shows relevance appraisals and the y-axis shows stress. The three lines represent the association between relevance (primary) appraisals and stress at low (−1 SD, blue dashed), average (mean, green solid), and high (+1 SD, orange dotted) levels of coping (secondary) resource appraisals; shaded areas denote 95% credible intervals. *Note.* (bs) = between-person variable; (ws) = within-person variable.

To examine whether individuals who generally appraised situations as more relevant (i.e., primary appraisals) and appraised fewer coping resources (i.e., secondary appraisals) report higher overall perceived stress, we predicted stress levels across the study period using the main effects and interaction of the between-person predictors. Individuals who in general appraised situations as more relevant experienced higher overall stress across the study period. Conversely, individuals with generally higher coping resource appraisals showed lower overall stress. In addition, there was an interaction between primary and secondary appraisals: individuals with generally low appraised coping resources exhibited a stronger association between appraised relevance of the situation and perceived stress levels than those appraising more coping resources. Fig 3 illustrates this pattern; in the left panel (Fig 3A), the relevance–stress slope is steeper when coping resources are appraised as low (−1 SD) and flatter when they are appraised as high (+1 SD), illustrating the moderating role of secondary appraisals.

Between-person, secondary appraisals showed the strongest association with overall stress (b = −.64, 95% HDI [−.75, − .53]), such that higher secondary appraisals were linked to lower stress. Higher primary appraisals showed the next-largest association (b = .24 [.12,  .35]). Both main effects fell entirely outside the ROPE and can therefore be regarded as practically meaningful. The interaction was considerably smaller (b = −.15 [−.25, − .06]); although statistically present, its practical relevance remained inconclusive. Across all effects statistical evidence was strong (all 95% HDIs excluding zero, PD ≥ 99.94%), yet this magnitude-based view shows that only the two main effects were practically meaningful (see Table 4). These conclusions did not change when age and sex were included as covariates.

#### RQ 2 - The “When”: Are moments with higher relevance appraisals and lower coping resource appraisals associated with higher momentary stress?

To examine whether timepoints at which individuals appraise situations as more relevant than usual (i.e., primary appraisals) and appraise fewer coping resources than usual (i.e., secondary appraisals) are associated with higher momentary stress, we predicted within-person stress using the main effects and interaction of the within-person predictors. As in the between-person model reported above, higher momentary relevance of a situation was associated with increased momentary stress levels. In contrast, higher appraisal of coping resources at a timepoint were associated with lower perceived stress. Further, an interaction indicated that the association between momentary appraised relevance of a situation and momentary perceived stress levels was stronger on occasions when individuals appraised fewer coping resources than usual. Fig 3 illustrates this pattern; in the right panel (Fig 3B), the relevance–stress slope is steeper when coping resources are appraised as low (−1 SD) and flatter when they are appraised as high (+1 SD), illustrating the moderating role of secondary appraisals.

Within-person, secondary appraisals again showed the strongest association with momentary stress (b = −.39, 95% HDI [−.44, − .34]), such that appraising more coping resources than usual was linked to lower momentary stress; with 0% of the posterior inside the ROPE, this effect can be regarded as practically meaningful. Higher momentary relevance appraisals showed a smaller association (b = .11 [.06,  .17]); although statistically present, its practical relevance remained inconclusive. The interaction was smallest (b = −.07 [−.10, − .03]) and can be considered practically equivalent to zero, as more than 95% of its posterior fell within the ROPE. Thus, while statistical evidence was strong across all effects (all 95% HDIs excluding zero, PD ≥ 99.98%), a magnitude-based view identifies only within-person coping resource appraisals as practically meaningful (see Table 4). These conclusions did not change when age and sex were included as covariates.

## Discussion

### General discussion

This study examined key assumptions of the Transactional Model of Stress using an EMA approach among adult samples. The Bayesian multilevel models showed clear associations between appraisals and perceived stress at both the within-person and between-person levels. Momentary higher levels in appraised relevance of a situation (primary appraisal) were associated with higher concurrent perceived stress levels, whereas momentary higher levels in appraised coping resources (secondary appraisal) were associated with lower perceived stress levels. Similarly, individuals who, on average, appraised situations as more relevant reported higher overall perceived stress, while those with generally higher coping resource appraisals showed lower overall perceived stress. These between-person effects may reflect relatively stable appraisal styles and suggest that individuals who generally perceive situations as more consequential and their coping resources as less adequate, may be particularly vulnerable to elevated stress levels and their health consequences. Multiple factors have been shown to play a role in explaining such interindividual differences in stress appraisals and stress responses. Besides situational factors, past experiences [51], or individual trait variables such as perceived stress reactivity [18], self-efficacy [52], or personality traits may explain why a person may generally appraise a situation as more relevant or coping resources as low. Neuroticism, for example, has been linked with increased threat appraisals, less successful coping and increased perceived stress [53,54].

Beyond these main effects, the models estimated the primary × secondary appraisal interaction. This interaction was statistically present at both levels (within-person and between-person) and pointed in the predicted direction: the association between relevance appraisals and stress was stronger when coping resources were appraised as low. The data thus support the Transactional Model of Stress prediction that the two appraisals do not act purely additively. Their combined magnitude, however, was smaller than that of the main effects. Between persons, the interaction was statistically present but did not cross thresholds for practical meaningfulness; within persons, the interaction was also statistically present yet practically negligible in size. The transactional interplay of appraisals was therefore directionally confirmed, while its practical magnitude was limited, particularly for moment-to-moment within-person fluctuations. These results not only concur with the assumptions of the Transactional Model of Stress, but are complemented by the Conservation of Resources Theory, which posits that a loss of resources or a perceived threat to resources can lead to perceived stress [55]. The stronger association between relevance appraisals and stress under conditions of low perceived coping resources suggests that appraisal-based and resource-based perspectives may provide complementary explanations of stress responses. A lack of coping resources may be appraised as particularly threatening in situations of high personal relevance [56]. In line with the appraisal main effects observed in our study, Fernández-Castro et al. (2022) found in a daily-diary study that higher primary and lower secondary appraisal were each associated with increased negative and decreased positive affect, although their models did not test an interaction between the two appraisals [43].

Taken together, these results support the central role that the Transactional Model of Stress assigns to cognitive appraisals, with both appraisals shaping stress responses, both moment-to-moment within individuals and in more stable differences between persons. In addition, the results add to previous studies showing that primary appraisals of threat or significance are positively associated with stress responses [57]. Within individuals, lower coping resource appraisals showed a practically meaningful impact in predicting *when* someone experienced higher-than-usual stress, while evidence on the practical impact of relevance appraisals was inconclusive. At the between-person level, both coping and relevance appraisals demonstrated practically meaningful associations with overall stress levels, underscoring their importance for explaining inter-individual differences in stress.

### Practical relevance and future research

These findings may help inform tailored stress-management interventions based on individual fluctuations of stress-related appraisals. As subjective stress often follows important real-life events, such as receiving a serious diagnosis, focusing on secondary appraisal and enhancing available coping resources seems a more realistic and fruitful approach instead of reducing the perceived relevance of a situation. For instance, Ecological Momentary Interventions (EMIs) could provide context-sensitive prompts increasing the cognitive availability of coping resources in situations appraised as more relevant or demanding than usual, potentially buffering subsequent stress. Integrating such context-sensitive prompts should be a next step in the development of the TrackYourStress mHealth platform. By reducing the intensity and frequency of such momentary stress spikes, such just-in-time-interventions may have potential benefits for both mental and physical health. In addition, EMA-based assessments of momentary stress levels provide an opportunity to inform personalized, context-sensitive stress management interventions. In this context, recommender systems that combine EMA-based stress screening with information on available mental health services may improve the accessibility, acceptance, and effective use of existing interventions by aligning support options with individuals’ current stress profiles [58].

Future research is needed to more specifically disentangle the dynamic, within-person sequences linking specific potential stressors, cognitive appraisals, coping responses, and their subsequent consequences. To keep participant burden as low as possible and to maintain adherence in such studies, assessments need to be as brief and efficient as possible. In line with this, an adapted short version of the widely used Perceived Stress Scale [59] has recently been introduced specifically for EMA research [40]. Incorporating multimodal assessments of stress in future studies may additionally strengthen the validity of stress measurements and enable researchers to examine how appraisals and coping efforts not only shape perceived stress but also influence physiological stress systems relevant to mental and physical health outcomes [47]. Such a multimodal stress assessment could include tools like the Mobile Photographic Stress Meter [60], passive sensors and other indicators from computational techniques [61–64] as well as physiological data [65,66]. Future versions of the TrackYourStress app could incorporate multimodal assessment methods to complement self-reported stress and appraisal measures. Promising modalities include activity- and mobility-related indicators, such as step count, walking activity, sedentary behavior, and mobility patterns, which could be obtained through platform-level health data interfaces such as Apple Health or Google Health Connect. In addition, passive smartphone-derived digital phenotyping indicators, such as touchscreen interaction and phone-use patterns, could serve as unobtrusive proxies for stress-related behavioral changes. Physiological indicators assessed via dedicated hardware or wearable sensors, such as heart rate, heart rate variability, electrodermal activity, or sleep parameters, could further capture stress-related physiological changes in daily functioning. Integrating these data streams with questionnaire-based assessments could enable a more comprehensive, temporally fine-grained, and ecologically valid assessment of stress in future app versions, as also suggested by recent work on combining EMA frameworks with passive sensing approaches [67]. However, privacy, user consent, technical feasibility, sensor availability, and the validity of passive indicators across individuals and contexts would need careful consideration.

### Limitations

This study has several limitations that should be considered when interpreting the results. First, internal validity is limited by the non-experimental design and by the fact that stress assessments were collected in naturalistic, everyday-life settings. Consequently, potential confounding variables could not be fully controlled [68]. For reasons of time economy, the TrackYourStress registration questionnaire assessed only a small set of sociodemographic characteristics; variables such as educational attainment, relationship status, income, and occupation were therefore unavailable and could not be included as covariates beyond age and sex.

Second, the sample was not randomly recruited. Participants were primarily drawn from universities and from personal networks of student recruiters, resulting in a convenience sample that is unlikely to be representative of the general population [69]. For reasons of anonymity, potential relationships between participants and recruiters were not assessed. Relatedly, because data collection was fully anonymized, participants’ student status and the timing of their assessments relative to the academic calendar (e.g., examination periods, teaching weeks, or semester breaks) were not recorded. As academic phases may substantially affect stress levels, this unmeasured temporal context represents a further limitation.

Third, external validity is constrained by differences between included and excluded TrackYourStress participants. Specifically, individuals included in the present study had, on average, lower stress levels at the initial stress report than those who did not meet the inclusion criteria, suggesting that individuals with higher stress levels dropped out early and are not covered in the current analysis.

Fourth, another limitation concerns potential biases in the time points of assessing momentary stress appraisals. Because participants completed the assessments at self-chosen time points, it is possible that they responded predominantly in particularly high- or low-stress moments, which may have influenced the results [70]. Relatedly, participants could choose between a fixed or random notification schedule; because this choice was not stored at the level of individual observations, it could not be examined as a potential moderator. As only the first daily assessment entered the analyses, however, the intra-day timing of prompts is unlikely to have affected the present results, and comparing sampling schedules directly remains a question for future research.

Fifth, appraisals and stress were assessed in parallel in the current study. Consequently, the present analyses do not allow conclusions about temporal precedence or causal ordering between appraisals and stress. Future research could therefore examine how appraisals at one time point (t) influence stress at subsequent time points (t + 1). Because time-lagged effects were not modeled here, future studies may benefit from explicitly capturing temporal dependencies and dynamic patterns in stress responses, for example by applying approaches such as dynamic time warping [71].

Sixth, our conclusions regarding momentary stress levels rely on a single-item measure of perceived stress, which may limit reliability [40]. In addition, because momentary stress and appraisals were each assessed with single items, the intraindividual variance component conflates true momentary fluctuation with occasion-level measurement error; the intraindividual variance should be interpreted with this caveat in mind. Future studies using multiple indicators per occasion should separate true momentary variance from measurement error. Relatedly, it should also be noted that measurements on Android and iOS smartphones may be perceived differently, as the user interface is never fully identical across operating systems.

Seventh, repeated daily assessments may increase participants’ awareness of stress-related experiences (reactivity), a common limitation of intensive longitudinal designs [37]. Such reactivity may operate in more than one direction, as repeated reflection could heighten awareness of both stress and one's own coping resources. A related concern is that participants could view a record of their own previous responses (e.g., stress ratings over time); although this feedback included no normative comparisons or recommendations, such self-monitoring can itself be reactive. Reassuringly, the model-based stress trajectories over time did not indicate a systematic increase (see Supporting Information S9 Text, S9 Fig), consistent with earlier TrackYourStress findings [17]. A systematic analysis of long-term within-person trends in stress and appraisals nonetheless remains a promising direction for future research.

## Conclusion

In sum, using an EMA approach, this study showed that both primary (relevance) and secondary (coping resource) appraisals were robustly and practically associated with perceived stress, from moment to moment within individuals as well as between individuals. Consistent with the Transactional Model of Stress, the two appraisals also interacted in the predicted direction, such that the association between relevance and stress was stronger when coping resources were appraised as low; the practical magnitude of this interaction was, however, limited. To our knowledge, this study provides the first EMA-based test to jointly examine primary (relevance) and secondary (coping resource) appraisals as well as their interaction as predictors of momentary stress while dissociating within- from between-person effects.

## Supporting information

S1 Text Illustration of the TrackYourStress app (App Screens). (DOCX)

S2 Text Receiving and Responding to Notifications. (DOCX)

S3 Text Registration Process. (DOCX)

S4 Text Registration Questionnaire. (DOCX)

S5 Text Notification Settings. (DOCX)

S6 Text Overview of Databases. (DOCX)

S7 Text Robustness of Findings. (DOCX)

S8 Text Sensitivity Analysis. (DOCX)

S9 Text Predicted Stress Levels Over Time Since Registration (Stress-Levels Over Tracking Duration). (DOCX)

S1 Table Results of Analysis Incorporating Recruitment Phase as Moderator. (DOCX)

S1 Fig Illustration of the TrackYourStress app (App Screens). (EPS)

S2 Fig Receiving and Responding to Notifications. (EPS)

S3 Fig Registration Process. (EPS)

S4 Fig Registration Questionnaire. (EPS)

S5 Fig Notification Settings. (EPS)

S6 Fig Duration in App. (TIF)

S7 Fig Number of Entries in App per Participant. (TIF)

S8 Fig Robustness of Findings. (TIF)

S9 Fig Predicted Stress Levels Over Time Since Registration (Stress-Levels Over Tracking Duration). (TIF)
