# Supplementary material for: From Who experiences stress to When It Occurs: Revisiting Lazarus’ transactional model of stress using an ecological momentary assessment framework
Source: PLOS Digit Health. 2026 Sep 15;5(9):e0001716. doi: 10.1371/journal.pdig.0001716 (PMC13577512; doi:10.1371/journal.pdig.0001716)
Supplement: Supplementary file 1 [file pdig.0001716.s001.docx]

**S1 Text**

*Illustration of the TrackYourStress app (App Screens)*

S1 Fig displays ten app screenshots: (a) the loading screen, (b) the profile page, (c) the settings page, (d) the language selection view, and (e) the list of submitted answer sheets. Panels (f)–(i) show the statistics page: (f) and (g) present the “Stress Reactivity: Perceived Stress Reactivity Scale” in two configurations; (h) shows the “Stress: Perceived Stress Scale”; (i) depicts the latest entry of the “Coping: Stress and Coping Inventory” as a radar chart; panel (j) shows its progression over time as a line chart; (k) displays the “Personality: Big Five Inventory–SOEP”; and (l) presents the “Daily: Stress & Coping” data over time as a line chart with a highlighted entry.
