# Supplementary material for: From Who experiences stress to When It Occurs: Revisiting Lazarus’ transactional model of stress using an ecological momentary assessment framework
Source: PLOS Digit Health. 2026 Sep 15;5(9):e0001716. doi: 10.1371/journal.pdig.0001716 (PMC13577512; doi:10.1371/journal.pdig.0001716)
Supplement: Supplementary file 2 [file pdig.0001716.s002.docx]

**S2 Text**

*Receiving and Responding to Notifications*

S2 Fig illustrates the complete process by which users receive and respond to scheduled follow-up questionnaires (daily, weekly, and monthly). The sequence begins with a push-notification reminder triggered automatically based on the predefined schedule. This appears both as a notification badge on the app icon and as lock-screen notification messages. Users can enter the app either by tapping the app icon or by opening a specific notification message. Both entry points lead to the questionnaire overview page, from which the user can access the relevant daily, weekly, or monthly questionnaire. When opened via a notification message, the app navigates directly to the corresponding questionnaire page. After completing the questionnaire, a confirmation popup indicates successful submission. From here, users can return to the questionnaire overview or adjust notification settings if needed. The flow can repeat until all due questionnaires have been completed.
