# Supplementary material for: From Who experiences stress to When It Occurs: Revisiting Lazarus’ transactional model of stress using an ecological momentary assessment framework
Source: PLOS Digit Health. 2026 Sep 15;5(9):e0001716. doi: 10.1371/journal.pdig.0001716 (PMC13577512; doi:10.1371/journal.pdig.0001716)
Supplement: Supplementary file 3 [file pdig.0001716.s003.docx]

**S3 Text**

*Registration Process*

S3 Fig outlines the complete onboarding process from first app launch through account creation, email verification, and initial app setup. Users start on the welcome page when not already logged in. From here, they may proceed to the login page or initiate registration. Account creation begins on the registration page and requires accepting the consent pop-up. If consent is declined or account creation fails, an error message is displayed; successful submission triggers an account-creation confirmation pop-up. The registration process continues with an email-confirmation step, during which users receive a registration-confirmation email. Clicking Complete Registration in this email finalizes the account, as illustrated by the registration-completion website in the diagram. Once the email is confirmed, users can sign in and proceed with onboarding. The onboarding stage includes granting notification permissions, after which the user is taken to the questionnaire overview page.
