# Supplementary material for: From Who experiences stress to When It Occurs: Revisiting Lazarus’ transactional model of stress using an ecological momentary assessment framework
Source: PLOS Digit Health. 2026 Sep 15;5(9):e0001716. doi: 10.1371/journal.pdig.0001716 (PMC13577512; doi:10.1371/journal.pdig.0001716)
Supplement: Supplementary file 4 [file pdig.0001716.s004.docx]

**S4 Text**

*Registration Questionnaire*

S4 Fig illustrates the full process of completing the registration questionnaire, which must be completed before the recurring questionnaires become available. The sequence begins on the questionnaire overview page, where the user selects the Registration Questionnaire. Upon entry, the app requests microphone and location permissions through system pop-ups. The user is then guided through a series of study-related information screens, demographic questions, and baseline assessment items. The diagram also shows example interactions within these pages, including text-input fields and multiple-choice questions. If items are left unanswered, a Missing Answers pop-up appears, prompting users to complete all required fields. Once the questionnaire is fully completed and submitted, a Submission Successful pop-up confirms completion. The user is then returned to the questionnaire overview page, where the daily, weekly, and monthly questionnaires are now unlocked and accessible.
