# Supplementary material for: From Who experiences stress to When It Occurs: Revisiting Lazarus’ transactional model of stress using an ecological momentary assessment framework
Source: PLOS Digit Health. 2026 Sep 15;5(9):e0001716. doi: 10.1371/journal.pdig.0001716 (PMC13577512; doi:10.1371/journal.pdig.0001716)
Supplement: Supplementary file 5 [file pdig.0001716.s005.docx]

**S5 Text**

*Notification Settings*

S5 Fig depicts the process for adjusting the app’s notification schedules. Users access these options by navigating from the settings page to the Notifications section, where all available schedules are listed. Selecting a schedule, or accessing it via Notification Settings in the Submission Successful popup, leads to a dedicated schedule-editing view that allows users to modify the number of notifications and their timeframe. Updated values are highlighted in green, while the active/inactive state of a schedule can be toggled, with the toggle action indicated in red. The figure also shows how a deactivated schedule is displayed. After changes are saved, the user is returned to the notification settings page. This process can be repeated for any editable schedule.
