# Supplementary material for: From Who experiences stress to When It Occurs: Revisiting Lazarus’ transactional model of stress using an ecological momentary assessment framework
Source: PLOS Digit Health. 2026 Sep 15;5(9):e0001716. doi: 10.1371/journal.pdig.0001716 (PMC13577512; doi:10.1371/journal.pdig.0001716)
Supplement: Supplementary file 6 [file pdig.0001716.s006.docx]

**S6 Text**

*Overview of Databases*

As shown in S6 Fig, participants varied substantially in the duration they tracked their stress (i.e., time from first to last entry in app). Participants in the first data collection wave typically remained active in the app for approximately one year. In contrast, tracking duration in the second data collection wave varied considerably, ranging from one day to as many as 516 days. Over the period in which participants remained active in the app, they also differed in the number of stress questionnaires they completed. As illustrated in S7 Fig, the number of entries in the app ranged from two to 36 or 126 in the first and second data collection wave, respectively.
