# Supplementary material for: From Who experiences stress to When It Occurs: Revisiting Lazarus’ transactional model of stress using an ecological momentary assessment framework
Source: PLOS Digit Health. 2026 Sep 15;5(9):e0001716. doi: 10.1371/journal.pdig.0001716 (PMC13577512; doi:10.1371/journal.pdig.0001716)
Supplement: Supplementary file 7 [file pdig.0001716.s007.docx]

**S7 Text**

*Robustness of Findings*

Because the number of app entries and the duration of stress tracking (i.e., time from first to last entry) varied substantially across participants, we evaluated the robustness of our findings across multiple thresholds for both data volume and tracking duration. This sensitivity analysis allowed us to assess whether the results were disproportionately influenced by a small number of high-contribution participants (e.g., individuals providing substantially more datapoints than others, such as the one outlier with 126 entries) or by participants who were especially persistent in stress tracking (which may indicate that they are more motivated or place high importance on stress tracking). To this end, we incrementally excluded participants with the highest number of entries or the longest tracking duration and re-estimated the models at each threshold. For example, we first reran the analyses including all participants, then excluded the participant with the highest number of entries (or longest duration of stress tracking), followed by those with the second-highest contribution, and so on, until the remaining sample size was too small for model convergence. These analyses were conducted using frequentist estimation, as fitting this series of 166 nested models in a Bayesian framework would have required prohibitively long computation times.

S8 Fig (A, upper plot) shows how significance of the six effects (main effects of resource and relevance appraisals and their interaction, each at the within- and between-person levels) as participants with the longest tracking durations were successively excluded. For the primary appraisals (i.e., relevance), the main effect of the remained significant until only 37 participants remained (20 days or fewer days between the first and last entry) for the within-person effect, and across all thresholds with the between-person effects. For primary appraisals (relevance), the within-person main effect remained significant until the sample was reduced to 37 participants (corresponding to 20 or fewer days between the first and last entry), while the between-person effect remained significant across all thresholds. For secondary appraisals (resources), the within-person main effect remained significant until 35 participants remained (17 or fewer days in the app), and the between-person effect was again robust across all thresholds. The interaction between primary and secondary appraisals remained significant until 68 participants remained (229 or fewer days in the app) for the within-person effect and until 64 participants remained (182 or fewer days) for the between-person effect.

S8 Fig (B, lower panel) shows the same set of analyses when participants were successively excluded based on the number of stress entries. For primary appraisals, the within-person main effect remained significant until 67 participants remained (15 or fewer entries), whereas the between-person effect was significant across all thresholds. For secondary appraisals, both within- and between-person main effects remained significant across all thresholds. The interaction effect remained significant until 73 participants remained (17 or fewer entries) for the within-person effect and until 55 participants remained (10 or fewer entries) for the between-person effect.
