# Supplementary material for: From Who experiences stress to When It Occurs: Revisiting Lazarus’ transactional model of stress using an ecological momentary assessment framework
Source: PLOS Digit Health. 2026 Sep 15;5(9):e0001716. doi: 10.1371/journal.pdig.0001716 (PMC13577512; doi:10.1371/journal.pdig.0001716)
Supplement: Supplementary file 8 [file pdig.0001716.s008.docx]

**S8 Text**

*Sensitivity Analysis*

To examine the robustness of our findings with respect to recruitment procedures, we conducted an exploratory analysis incorporating recruitment phase as a moderator. Specifically, we introduced a dummy-coded variable (0 = recruitment phase A; 1 = recruitment phases B and C) and tested its involvement in lower- and higher-order interaction terms with the primary predictors. Please note that recruitment phases B and C were combined because participants in both phases accessed the study via the app store, and the available data did not allow us to distinguish between these recruitment strategies. Consequently, any potential differences between phases B and C could not be modeled separately.

As seen in S1 Table, there was sufficient evidence for a practically meaningful effect indicating that participants recruited during phases B and C reported higher average stress levels across the study period that participants recruited during phase A (i.e., Between-Person Model). Importantly, findings reported in the main manuscript remained robust. Specifically, the main effects of primary and secondary appraisals, as well as their interaction, on average stress levels were preserved (i.e., practically meaning full effects for primary and secondary appraisals as well as a statistically significant effect for their interaction effect), and there was no sufficient evidence that these effects dependent on recruitment phase (i.e., 95%HDIs of two- and three-way interactions with the recruitment phase included zero). At the same time, less than 95% of the evidence fall within the ROPE for the interaction terms involving the recruitment phase, which also precludes concluding practical equivalence (i.e., evidence for no effect of recruitment phase).

For the Within-Person Model, main and interaction effects for primary and secondary appraisals were also consistent with those reported in the main manuscript, and there was sufficient evidence to conclude that the recruitment phase had no effect on momentary stress levels (i.e., more than 95% of evidence in the ROPE). This is expected, as the within-person specification isolates intra-individual fluctuations (i.e., deviations from an individual’s typical stress level) and, by design, removes between-subject variability, including differences attributable to recruitment procedures. Evidence for interactions with recruitment phase remained inconclusive (i.e., no evidence for a statistical meaningful effect but also no sufficient evidence for an effect that is practically equivalent to zero).
