# Supplementary material for: From Who experiences stress to When It Occurs: Revisiting Lazarus’ transactional model of stress using an ecological momentary assessment framework
Source: PLOS Digit Health. 2026 Sep 15;5(9):e0001716. doi: 10.1371/journal.pdig.0001716 (PMC13577512; doi:10.1371/journal.pdig.0001716)
Supplement: Supplementary file 9 [file pdig.0001716.s009.docx]

**S9 Text**

*Predicted Stress Levels Over Time Since Registration (Stress-Levels Over Tracking Duration)*

S9 Fig is a spaghetti plot showing predicted stress levels over time since registration. Grey points indicate the raw data. The black line represents the fixed effect of time, while the coloured lines show individual-level predicted trajectories from the Bayesian mixed-effects model.
