# Supplementary material for: From Who experiences stress to When It Occurs: Revisiting Lazarus’ transactional model of stress using an ecological momentary assessment framework
Source: PLOS Digit Health. 2026 Sep 15;5(9):e0001716. doi: 10.1371/journal.pdig.0001716 (PMC13577512; doi:10.1371/journal.pdig.0001716)
Supplement: Supplementary file 10 [file pdig.0001716.s010.docx]

**S1 Table**

*Results of Analysis Incorporating Recruitment Phase as Moderator*

|  | Between-Person Model (the „Who“) | | | |  | | | Within-Person Model (the „When“) | | | | | | |  |
| --- | --- | --- | --- | --- | --- | --- | --- | --- | --- | --- | --- | --- | --- | --- | --- |
| *Predictors* | *b* | *95%HDI* | *PD* | *ROPE* |  | *b* | | | *95%HDI* | | *PD* | | ROPE | |  |
| Primary Appraisals | **.21** | **[.08, .34]** | **99.88%** | **5%** |  | | .12 | | | [.05, .18] | | 99.98% | | 29.07% | |
| Secondary Appraisals | **-.63** | **[-.75, -.49]** | **100%** | **0%** |  | | **-.39** | | | **[-.45, -.33]** | | **100%** | | **0%** | |
| Recruitment Phase | **.58** | **[.36, .80]** | **100%** | **0%** |  | | 0 | | | [-.07, .07] | | 50.20% | | 99.38% | |
| Primary × Secondary | -.13 | [-.24, -.01] | 98.59% | 31.91% |  | | -.07 | | | [-.11, -.04] | | 99.98% | | 90.06% | |
| Primary × Recruitment | .17 | [-.05, .39] | 92.97% | 27.28% |  | | -.02 | | | [-.17, .11] | | 63.35% | | 81.64% | |
| Secondary × Recruitment | .16 | [-.06, .37] | 92.27% | 28.92% |  | | -.01 | | | [-.12, .14] | | 52.91% | | 87.13% | |
| Primary × Secondary × Recruitment | -.07 | [-.28, .12] | 74.64% | 57.67% |  | | .05 | | | [-.04, .14] | | 86.94% | | 85.42% | |

*Note*. Bold values denote effects considered practically meaningful.
